# Eye movements during listening reveal spontaneous grammatical processing

**DOI:** 10.3389/fpsyg.2014.00410

**Published:** 2014-05-21

**Authors:** Stephanie Huette, Bodo Winter, Teenie Matlock, David H. Ardell, Michael Spivey

**Affiliations:** ^1^Department of Psychology, University of MemphisMemphis, TN, USA; ^2^Cognitive and Information Sciences, University of CaliforniaMerced, CA, USA; ^3^Quantitative and Systems Biology, University of CaliforniaMerced, CA, USA

**Keywords:** language, eye movements, linguistic theory, embodied cognition, perceptual simulation

## Abstract

Recent research using eye-tracking typically relies on constrained visual contexts in particular goal-oriented contexts, viewing a small array of objects on a computer screen and performing some overt decision or identification. Eyetracking paradigms that use pictures as a measure of word or sentence comprehension are sometimes touted as ecologically invalid because pictures and explicit tasks are not always present during language comprehension. This study compared the comprehension of sentences with two different grammatical forms: the past progressive (e.g., *was walking*), which emphasizes the ongoing nature of actions, and the simple past (e.g., *walked*), which emphasizes the end-state of an action. The results showed that the distribution and timing of eye movements mirrors the underlying conceptual structure of this linguistic difference in the absence of any visual stimuli or task constraint: Fixations were shorter and saccades were more dispersed across the screen, as if thinking about more dynamic events when listening to the past progressive stories. Thus, eye movement data suggest that visual inputs or an explicit task are unnecessary to solicit analog representations of features such as movement, that could be a key perceptual component to grammatical comprehension.

## Introduction

The capacity to think about past, present or future events is a fundamental cognitive ability (Zacks and Tversky, 2001). Language taps into this capacity by directing how one thinks of a particular event (Givón, 1992). Grammatical aspect has the ability to specify fine-grained temporal differences that are implied. With the sentence *John was going to the store* (past progressive) the unfolding of an event is emphasized, whereas with the sentence *John went to the store* (simple past) the end state is emphasized. The progressive is a linguistic structure that focuses on the dynamics of an event, which is construed as happening in the moment of a story's time frame. Some have invoked a cinematic analogy to describe the effects of the progressive, likening the progressive to a movie (Kruisinga and Erades, 1955). In contrast, the simple past de-emphasizes the dynamic, durative or repetitive aspects of a described situation, focusing merely on a static end point. This distinction is supported by linguistic research on aspect (Comrie, 1976; Dowty, 1977; Langacker, 1982), as well as psychological research on aspect (Magliano and Schleich, 2000; Madden and Zwaan, 2003; Matlock, 2011). The work to date has shown these effects in tasks where the participant formulates a response and is given an explicit task, but little is known about whether these kinds of subtle perceptual effects have an influence in the absence of an explicit task or goal.

Behavioral and electrophysiological evidence demonstrate that sentences with progressive aspect activate more richly detailed event knowledge, for instance, details about location and the participants in a scene (Carreiras et al., 1997; Ferretti et al., 2007). The progressive's emphasis on the ongoing nature of events has been argued to draw attention to the motion of described actions (Anderson et al., 2008, 2013) and to facilitate congruent motor movement (Bergen and Wheeler, 2010). These properties are also reflected in co-speech gestures, which are more extended or iterative in the context of progressive language (Duncan, 2002; Matlock et al., 2012; Parrill et al., 2013). Non-progressive forms, on the other hand, have been found to direct attention to the completion of an event and the static endpoint of a movement (Magliano and Schleich, 2000; Madden and Zwaan, 2003; Athanasopoulos and Bylund, 2013). The distinction between these two forms has important real-world consequences for how people interpret actions and ultimately how it affects attitudes and perceptions, including voting preferences (Fausey and Matlock, 2011; Matlock, 2012) and eyewitness testimony (Matlock et al., 2012).

Most of this work on the link between language and sensorimotor representations has used explicit tasks that constrain the participant's response by the pictures used or the response options available. Thus one criticism that may arise is that participants may be prompted to simulate perceptual features correlated with these kinds of sentences, because their response options are often perceptual (see discussion in Pecher et al., 2009). This also bears on work utilizing the visual world paradigm: for example in a task where participants must click on a picture when the word is spoken (e.g., the spoken word “cat,” presented in concurrence with pictures of a cat, a dog, and two unrelated items), it is conceivable that eye movements to the semantically related picture of a dog were prompted by its concurrent presence. It could be hypothesized that in the absence of this kind of concurrent presentation, activation of corresponding visually and semantically related information may not be present. Many studies have used this kind of paradigm to investigate how and what speech information is activated during spoken word recognition, but it is not known how the task itself affects processing (see Huettig and Altmann, 2005 for this demonstration of semantic competition in the visual world paradigm).

Eye-movement data have offered crucial guidance for theories of language processing in various specific contexts, such as reading (Rayner, 1998; Spivey and Tanenhaus, 1998), integrating diagrams with text (Hegarty and Just, 1993), following spoken instructions (Tanenhaus et al., 1995), and engaging in directed mental imagery in the absence of visual cues (Spivey and Geng, 2001; Altmann, 2004). Eye-tracking is an unobtrusive measure that collects multiple data points per experimental trial (saccadic eye movements and fixations to locations on a screen). However, experiments using this technology are typically not so unobtrusive, because they involve tasks that require explicit judgments on visual or linguistic stimuli and as such, task demands. Our experiment addresses this concern by doing an eye-tracking study without visual referents. We examine a behavior that is constantly in flux—the movements of the eye—, as a function of auditory linguistic stimuli only. In other words, we look at the effects of language comprehension on eye movements in the absence of task-relevant visual stimulation.

Previous studies have shown that language comprehension interfaces with motion processing, but stimulating the motor cortex can also impair comprehension (Hauk et al., 2004; Pulvermüller, 2005; Zwaan and Taylor, 2006; Bergen and Wheeler, 2010). Further, eye movements on a blank screen reflect the spatial content of verbally described scenes (Spivey and Geng, 2001). Importantly, many theories argue that higher level language processing is so far removed from perception as to be symbolic or amodal in nature (e.g., Mahon and Caramazza, 2008). Demonstration of a link between low-level sensorimotor features and higher level language processing has not yet been shown in the absence of a perceptual prompt, and thus this research will examine eye movements on a blank screen as a measure of what we operationally call *spontaneous processing*. Under this, we understand behavior and processing that occurs in the absence of an explicit task or concurrent visual referents to spoken words.

Importantly, the lack of visual and task constraints in this study is necessary, as the visual scene will not change or influence eye movements. Thus, the time course of how motion information is processed in language (in this case grammatical aspect, which—according to linguists—should change the semantic representations of space and time) may be a source of information that cascades to other areas of visual and motor cortex in meaningful ways that are not directly linked to a task constraint. Methodologically, most cognitive research takes place in a very constrained task or goal-oriented context, and the current methodology provides a window into information flow in the absence of these experimental constraints. Thus, our experiment tries to mimic situations that frequently arise in normal conversation, where people often use abstract language and references to non-present objects and ideas. Our study provides a framework for demonstrating in what ways language may interact in conditions similar to listening to a lecture or having a casual conversation.

Based on the above-mentioned linguistic analyses of aspect and experimental studies, we predicted that eye movements should be noticeably shorter and more widely dispersed in the past progressive. This prediction is motivated in the following way: If a series of past progressive sentences such as “He was going” induces focus on the dynamics of movement (Anderson et al., 2008, 2010; Matlock et al., 2012), more movement iterations (Duncan, 2002; Parrill et al., 2013), more vivid mental simulation (Bergen and Wheeler, 2010), more action in a given time period (Matlock, 2010, 2011) and the middle of a path or action (Morrow, 1985, 1990; Magliano and Schleich, 2000; Madden and Zwaan, 2003; Athanasopoulos and Bylund, 2013), this should lead to more thoughts of motion and action, and conceptualizations of motion in language processing have been shown to elicit more eye movements (Richardson and Matlock, 2007). Further, there is evidence that the perception of motion is tightly linked to eye movement areas, where motion strength and duration was directly proportional to the amplitude of an elicited saccade in primate oculomotor cortex (Gold and Shadlen, 2000). This suggests that even before a saccade is planned and launched, at least perceptually, that information about motion has already been shared with eye movement areas.

By contrast, a series of simple past sentences (“He went”) focuses on the static, completed end-state of the described event resulting in longer fixation times as if staring at a static object or scene. In addition to the temporal signal, the spatial pattern should reveal a larger area of scanning in the past progressive than in the simple past. This is predicted because cognitive linguists assume that the progressive creates an “unbounded” construal of an event, where a scene is viewed from within the scene (Langacker, 1982), whereas a sentence involving a simple past construction is thought to involve a “bounded” construal of an event, viewed externally. In contrast, as the simple past has been likened to viewing a static photograph (Kruisinga and Erades, 1955) and been found to emphasize the end point of a scene (Magliano and Schleich, 2000; Madden and Zwaan, 2003; Athanasopoulos and Bylund, 2013), we would expect more fixations on the same point in this condition. Just as the eyes move more when viewing motion and move more when listening to sentences invoking motion (e.g., Richardson and Matlock, 2007), the emphasis on more movement, longer durations or more repetition in the case of the past progressive, if encoded at least partly as low-level sensorimotor information, should elicit these shorter and more fleeting fixations to more different points on the screen.

## Methods

### Participants

Sixty-three right-handed native English-speaking participants were monitored and responses and fixations were recorded with an Eyelink II eye-tracking system. The study was approved by the University of California, Merced's IRB and informed consent was obtained for each participant. They received extra credit for participation in a social sciences course at the University of California, Merced. All participants had normal or corrected-to-normal vision and reported having no hearing problems or language deficiencies.

### Materials

Stories were manipulated with respect to grammatical aspect. Each story consisted of three to four sentences each, for example “John was on a bike ride yesterday. After he **sped / was speeding** across the valley, he **climbed / was climbing** a mountain range. Then he **pedaled / was pedaling** along a river and finally, he **coasted / was coasting** into a campground.” Each story was recorded by the same trained linguist (male, native English speaker). The final stimulus was chosen on the basis of the most similar prosody and intonation for both simple past and past progressive versions. The Supplementary Material contains all stimuli in both forms. There was a slight difference between the total duration of the past progressive stories (186.9 s) and the simple past stories (174.4 s). To account for the possibility that more fixations in the progressive condition were the result of this durational difference, we added 2 s of silence at the end of each sentence or clause boundary. This provides an equal time-window for the analysis across both conditions to analyze, ruling out the possibility that more samples in the past progressive condition could account for the results obtained, as well as provide a window of time to see if this effect persists for a brief duration after the sentence has ended. The duration of the silence was also found to sound like a natural speech pause. The sound start and end events were recorded along the eye movement data to allow parsing time into three windows: total time, linguistic stimulus only, and period of silence only. While there are other ways to control for time differences, this method has the advantage of allowing us to determine whether any effects of grammatical aspect persist after sentence completion.

### Procedure

The eye movement data were recorded at 500 Hz. Participants completed a picture-viewing task unrelated to the main experiment. In this task there were two pictures: one of a city street with stop lights, cars, trees and a clock tower, and the other a picture of a dog with a tennis ball. These two pictures were presented 3 times each in the same order for all participants. The first presentation had a gray square mask at their point of fixation, and followed their fixations in real time. The second presentation blurred everything but their point of fixation, again following their point of fixation as their eyes moved around the picture. The third presentation grayed out the background, but the picture could be seen at the point of fixation (similar to the blurring effect but entirely grayed out in the periphery). After completing that task, participants were informed they would next hear a set of stories that would help them forget the pictures they had just viewed. Before the task, participants were told to keep the eyes open and look at the screen so that recalibration of the eye tracker would not be necessary. Participants then listened to 22 short vignettes in either the past progressive or the simple past condition. A total of 31 of the participants were randomly assigned to the progressive condition, and a total of 32 to the simple past condition. There was no explicit task while listening over the headphones, and the “visual world” in front of them was simply a blank white screen. After the end of the experiment, participants were asked what they believed the nature of the task was. No one reported having a hypothesis that grammar was the manipulation, or that they predicted a magnitude difference in eye movements. Most naïve hypotheses about the nature of the experiment included the first viewing task that was not a part of the experiment.

It is worth noting that we chose to conduct a between subject experiment to complement previous within subject experiments (e.g., Parrill et al., 2013), which make the experimental manipulation salient and invites the problem that people might become aware of aspectual distinctions, potentially leading to strategic or metalinguistic responses. In general, it is important to conduct both within and between subject experiments on the same topic (the same argument has been made in the reasoning literature, see Stanovich and West, 2008; and for metaphor, see Winter and Matlock, 2013). For aspect in particular, between subject experiments have shown convergent results that are similar to within subject experiments (see Matlock, 2010, 2011; Fausey and Matlock, 2011). Given that assignments to the past progressive and simple past conditions were made randomly by the experimental procedure, the inferential statistics reported below allow us to be confident that differences found between conditions are not likely to be the result of differences between individuals (i.e., an independent samples *t*-test is precisely designed to show that differences between two groups is not due to unlucky sampling of individuals). Moreover, we checked whether there were fixation duration differences between people in the past progressive and the simple past conditions in the picture viewing portion that preceded the experiment, and none were observed (*p* > 0.1). This suggests that the results reported below are due to the grammatical manipulation in the auditory stimuli rather than due to individual differences.

## Results

**Figure 1:**
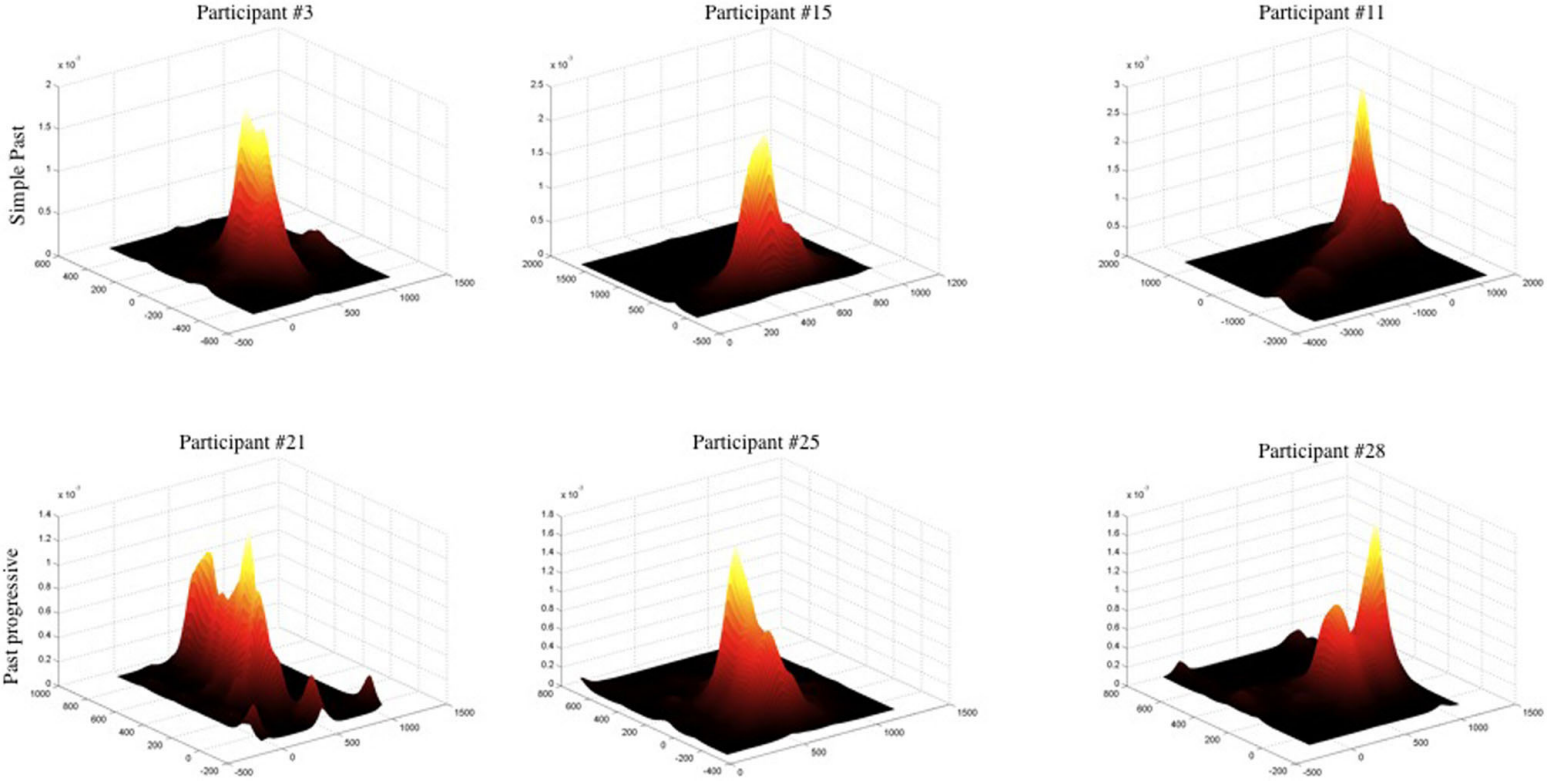
**Representative sample of individual fixation patterns, revealing a narrower spread of eye movements in the simple past condition (top row) as opposed to the past progressive condition (bottom row)**. The vertical axis shows total time spent fixating a given x, y location on the blank white screen. Each plot was *z*-scored. To more accurately represent dense areas, bivariate data was smoothed via a procedure appropriate for skewed data sets, for visual presentation only.

Unless otherwise noted, reported results refer to the full period of each item (sentence period + the following silence). Other periods will be considered below. The first set of analyses sought to describe the characteristics of the spatial differences in the data. Participants showed differential spatial distributions of their eye movements as a function of grammatical aspect. In the non-progressive aspect condition (with simple past tense sentences), participants tended to fixate their eyes on the central portion of the blank screen throughout the experiment, with few looks to the periphery; see Figure 1, upper row. By contrast, in the progressive aspect condition (with past progressive sentences), participants moved their eyes around in a wider area; Figure 1, lower row. To standardize the comparison of eye-movement dispersion across these two conditions, each participant's fixation data were individually *z*-scored so that means were aligned and distributional characteristics were not an artifact of averaging variant means. Subsequently, real-time fixation data were pooled into cumulative distributions for progressive and non-progressive conditions. When the average time spent fixating each x, y pixel (i.e., dwell time) in the past progressive condition is subtracted from the average dwell times for every x, y pixel in the simple past condition, the differences are found in the center of the distribution around the mean. This results from a substantial difference in the kurtosis (“peakedness”) of the two distributions. Cumulative kurtosis measures were higher in the simple past condition (x-axis: 11.2, y-axis: 11.8) than in the past progressive condition (*x*: 8.4, *y*: 7.3). Moreover, a two-sample Kolmogorov-Smirnov test along x and y screen dimensions revealed that non-progressive and progressive distributions differed significantly from one another along the x-axis (*D* = 0.02056, *df* = 62, *p* < 0.0001) and the y-axis (*D* = 0.0599, *df* = 62, *p* < 0.0001).

More detailed distributional analyses showed that the non-progressive simple past condition was associated with a greater proportion of sentences during which a participant fixated in only one location (using Monte Carlo simulated *p*-value, χ^2^ = 56.1574, *df* = 1, *p* < 0.0001), and also a greater proportion of sentences during which a participant fixated in no more than two locations (χ^2^ = 75.1473, *df* = 1, *p* < 0.0001). In the progressive condition, participants swept out more across the visual plane as measured by Area of the Convex Hull (ACH) of standardized eye movements (truncating outliers with standardized ACH > 30 or ACH = 0, comparing medians by condition, Wilcoxon test *W* = 1949210, *p* < 0.0001), and moved their eyes for greater total distances as measured by Total Path Length (TPL) of standardized eye movements (excluding TPL = 0; comparing medians by condition, Wilcoxon test *W* = 2265549, *p* < 0.0001). All of these measures suggest that the eyes covered a wider area when listening to progressive sentences, and that more distinct points on the screen were fixated.

**Figure 2:**
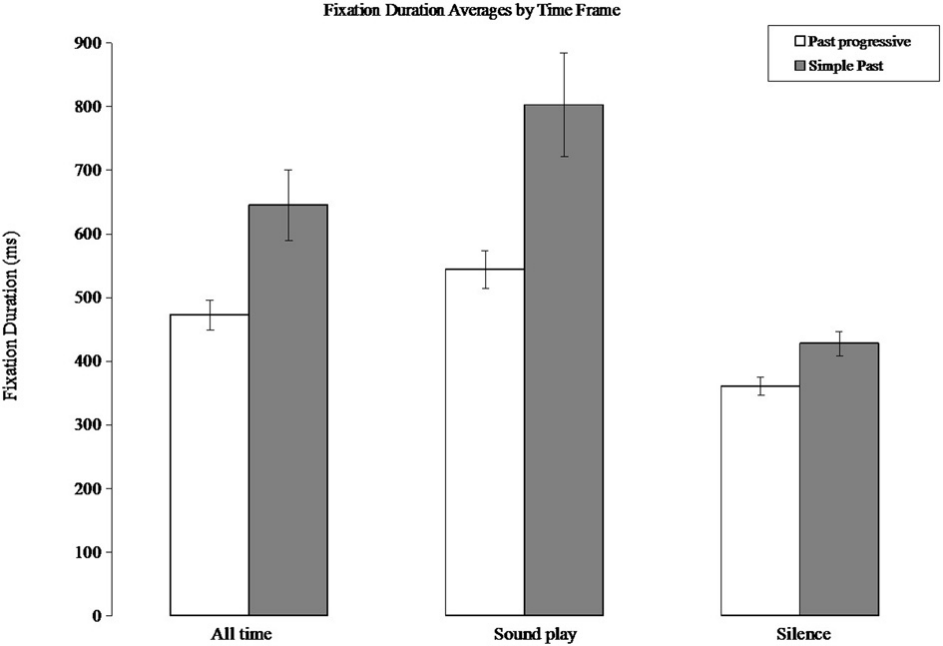
**Fixation duration averages in simple past and past progressive conditions by time frame**. “All time” is pooled across the sound playing and silence data. The average fixation durations are shorter in the progressive condition where the grammar implies an emphasis on motion. Error bars are s.e.m.

Not only did participants move their eyes around in a wider dispersion in the progressive aspect condition, they also produced briefer fixations in order to achieve that broad distribution. In the past progressive condition, fixation durations averaged 473 ms, whereas in the simple past (non-progressive) condition, fixation durations averaged 645 ms (independent samples *t*-test: *t*_(61)_ = 2.8, *p* = 0.006). Compared to other studies on grammatical processing or eye movements during language comprehension this is a large difference in fixation times. Thus, something as seemingly automated as how long the eyes remain stable in between saccadic eye movements is substantially influenced by the temporal emphasis implied by the grammar. This difference is present during the time segments in which speech is being played (past progressive mean: 543 ms; simple past mean: 802 ms; *t*_(61)_ = 3, *p* = 0.004). Importantly, this difference also persists when analyzing only the two-second silences in between each sentence (past progressive mean: 360 ms; simple past mean: 428 ms; (*t*_(61)_ = 2.7; *p* = 0.008). See Figure 2. Note that the mean difference in fixation duration between the two conditions was much larger while the speech was playing (simple past: 802 ms, past progressive: 544 ms) than during the period of silence (simple past: 428 ms, past progressive: 361 ms). This speaks to the importance of the linguistic information in triggering oculomotor processes, as it suggests that eye movements may be more affected by grammar when they are co-occurring with these grammatical properties, similar to what has been observed to motor congruence effects in sentence processing (Taylor and Zwaan, 2008). However, it is important to emphasize that the effect was significant in all three time windows.

## Discussion

As our results demonstrate here, there is nothing passive about passive listening: the eyes are actively moving in a way that reflects subtle grammatical differences in the linguistic input. The actual eye movement patterns are in line with what was predicted based on linguistic analyses of aspect and previous experimental work: past progressive appears to emphasize the ongoing motion of described actions and the details of described events, such that sentences with past progressive induce eye movements to be shorter and more dispersed—even while viewing a completely blank screen. These results suggest a smooth cascading of information from language processes in the brain all the way to oculomotor processes (Tanenhaus et al., 1995).

Moreover, these results provide converging evidence for the view that grammatical aspect changes the dynamics with which described events are construed. Previous work has shown that aspect changes event construal, but it has used tasks that involve pictures (Madden and Zwaan, 2003), maps (e.g., Morrow, 1985), constrained motor responses (Bergen and Wheeler, 2010) or limited response options (e.g., Matlock and Fausey, 2010; Matlock, 2011). This task does not constrain participants in a similar fashion. This coincides nicely with the results of Matlock et al. (2012), where participants watched video-taped events and were prompted with either “What happened?” (simple past) or “What was happening?” (progressive) before describing what they had seen. They spontaneously provided more action details and performed more action gestures when prompted by the progressive question. While that study showed how grammatical aspect can affect re-telling, the present study shows how it can influence eye movements during passive listening, an even less constrained task.

It is important to point out that we did not control for certain semantic factors that relate to aspect. For instance, our stimuli included a combination of atelic verbs (events with no explicit goal, such as “hang out”) and telic verbs (events with a specific goal and end state, such as “prepare a meal”). They also included verbs of short duration and long duration (e.g., yell at the umpire, climb a mountain range). Although some linguistic theories have addressed interactions between grammatical aspect and verb semantics (for review, see Sasse, 2002), there is currently only limited experimental work on this topic (Yap et al., 2009; Becker et al., 2013).

There are some important linguistic differences between past progressive and simple past that could play a role in these findings, though arguably do not account for the pattern of results found here. First, past progressive forms contain more morphemes than do simple past verb forms. Second, progressives forms are reported to be less frequent in English than non-progressive forms (e.g., Biber and Reppen, 2002). Considerable work in the eye-tracking reading literature has conclusively demonstrated that longer words and less frequent words elicit longer fixation durations when those words are being read (Rayner, 1998). Note, however, that the words in this task are delivered auditorily and the eyes are busy fixating a blank computer screen. Unlike the case in reading, in this auditory listening task the fixations themselves are not yoked to the delivery of each word in the linguistic input. Therefore, in this paradigm, there is no coherent prediction that word length or frequency makes for the duration of fixations or the spatial dispersion of them.

The time course of the effects we observed provides clues to the validity of the alternative explanations: It is not clear that any one of the alternative accounts (number of morphemes, frequency) predicts fixation duration differences both for the sentence presentation period *and* the silent period after the sentence presentation. However, an account based on the involvement of motor and sensory systems during language comprehension predicts both effects, because language-induced simulation effects have been found during incremental sentence processing (Zwaan and Taylor, 2006; Taylor and Zwaan, 2008; Sato et al., 2013) as well as at the end of sentence processing (Stanfield and Zwaan, 2001; Glenberg and Kaschak, 2002; Zwaan et al., 2002). Finally, it is not clear that the alternative accounts make both spatial and temporal predictions in the direction that we observed—which, as discussed above, follow from linguistic theory of grammatical aspect.

A final concern relates to the between subjects nature of our experimental design. Could the present effects be a consequence of individual differences? As argued above, this is not likely. First, the appropriate inferential statistics for between subjects designs tell us that the differences between groups are unlikely due to chance sampling, given that our assignment to conditions was random. Second, as reported above, we found no differences in the preceding task where the grammatical manipulation was not introduced yet. Third, the findings here conceptually mirror previous experiments on aspect that have used between subjects manipulations, such as Fausey and Matlock (2011), Matlock (2011), and Matlock et al. (2012). Why would individual differences happen to pattern along the same lines of these past results along multiple measures, if not because of grammatical aspect?

In general, many between subjects replications find similar effects that have previously been achieved with within subjects designs only (e.g., Stanovich and West, 2008; Winter and Matlock, 2013) with the added advantage that results are unlikely to be due to carry-over effects or to awareness of experimental manipulation. Choosing a between subjects design is furthermore closely connected to this study's main goal of highlighting language comprehension in more naturalistic processing situations because it prevents participants from discovering the grammatical manipulation. Because we found results that are consistent with cognitive linguistic views of aspect, the present results provide additional, converging evidence for the involvement of sensory and motor systems in understanding grammar on top of previous within and between subjects experiments on this topic.

Overall, the results are generally consistent with theoretical accounts of real-time language processing that emphasize the role of sensorimotor properties in linguistic content (Zwaan and Taylor, 2006; Meteyard et al., 2007; Barsalou, 2009). These results also begin to hint at the underlying mechanisms of information flow in the absence of a goal based context. Because language arrives and leaves so quickly, the use of perceptual-motor primitives associated with certain language contexts would allow for rapid comprehension of implied and related ideas, and the memory trace of this simulation could potentially help build a discourse context. Thus, the process of comprehending language involves accessing previously learned perceptual-motor information, and need not be solicited by explicit, concurrent visual stimuli.

The methods here provide a foundation for beginning to investigate the link between language and vision in a context that may not include visual referents. In addition, the processing of any kind of auditory stimuli (e.g., music, rhythm, sound) could be used and measured using this blank visual world paradigm. Many analyses on the time course and spatial characteristics could be done on eye movement data to find what the structure of the data reveals about the principles of processing.

These findings are in line with previous research that shows how described events with detailed spatiotemporal parameters involve sensorimotor systems of the brain (Hauk et al., 2004; Pulvermüller, 2005; Meteyard et al., 2007). Here we demonstrated that grammar affects a whole suite of different measurements connected to eye movements in a situation that minimizes task demands and mirrors real-world passive listening circumstances. This provides compelling evidence in favor of the view that the neural circuitry devoted to language is tightly connected with perceptual and motor areas of the brain and that grammar (such as progressive aspect) taps into sensorimotor representations and is able to modulate them.

This work also bears on theories of representation and argues against the idea that semantic networks are amodal or symbolic. Grammar is traditionally an “abstract” representation that does not have one to one mappings from perception to meaning. However, language learning always occurs in context, and the grammar used may over time be used in a context that has more features, or be used to augment certain features. In the case of the past progressive, one of the features that may have been accrued over time is movement, perhaps in addition to other not quite so easily identifiable perceptual features. During comprehension, this part of the grammar is activated along with the features of the words themselves. Previous work has shown the influence of perceptual features at the word level, but this work takes it one step further into demonstrating the how grammar is grounded in real-world features. Even the abstract parts of language such as grammar, and perhaps even prepositions, syntax, or other seemingly amodal levels of language processing may be more perceptually grounded than previously thought.

## Author contributions

All authors contributed to the conception, design, acquisition, analysis or interpretation of data, drafted or revised the manuscript critically for intellectual content, approved its final version, and agree to be accountable for all aspects of the work in ensuring that questions related to the accuracy or integrity of any part of the work are appropriately investigated and resolved.

### Conflict of interest statement

The authors declare that the research was conducted in the absence of any commercial or financial relationships that could be construed as a potential conflict of interest.
