# Supplementary material for: Eye movements during listening reveal spontaneous grammatical processing
Source: Front Psychol. 2014 May 21;5:410. doi: 10.3389/fpsyg.2014.00410 (PMC4033228; doi:10.3389/fpsyg.2014.00410)
Supplement: Supplementary file 1 [file DataSheet1.DOCX]

**Supplementary Material**

Each stimuli is listed in both simple past and past progressive. Differences between the two stimuli are in bold.

1. Peter was on the road last week.  He **was driving** to Florida to see his friend.  He was **staying** in cheap motels and **eating** in diners.  He **was using** his mother's credit card.

Peter was on the road last week.  He **drove** to Florida to see his friend.  He **stayed** in cheap motels and **ate** in diners.  He **used** his mother's credit card.

2. John was on a bike ride yesterday.  After he **was speeding across the valley,** he **was climbing** a mountain range.  Then he **was pedaling** along a river and finally, he **was coasting** into a campground.

John was on a bike ride yesterday.  After he **sped across the valley**, he **climbed** a mountain range.  Then he **pedaled** along a river and finally, he **coasted** into a campground.

3. Larry was at the park last night.  He **was playing** baseball with friends.  He **was throwing** curve balls.  He **was hitting** singles and doubles.  He was **yelling** at the umpire.

Larry was at the park last night.  He **played** baseball with friends.  He **threw** curve balls**.** He **hit** singles and doubles.  He **yelled** at the umpire.

4. John was stuck at the airport yesterday.  First he **was talking** to an airline attendant, then he **was sitting** in Starbucks.  He **was calling** friends on his cell phone.  He **was napping** in a lounge.

John was stuck at the airport yesterday.  First he **talked** to an airline attendant, then he **sat** in Starbucks.  He **called** friends on his cell phone.  He **napped** in a lounge.

5. Carl was at a mall yesterday.  First he **was hanging** out with friends at the food court.  Next he **was searching** for new shoes.  Then he **was buying** his dad a new CD.

Carl was at a mall yesterday.  First he **hung** out with friends at the food court.  Next he **searched** for new shoes.  Then he **bought** his dad a new CD.

6. Jim was at a campground last weekend.  He w**as unloading** his car. Then he **was putting** up a tent.  He **was cutting** wood and **building** a fire.

Jim was at a campground last weekend.  He **unloaded** his car.  Then he **put** up a tent.  He **cut** wood and **built** a fire.

7. Todd was at a wedding reception last night.  He **was eating** cake and **drinking** wine.  He **was toasting** the bride and groom and in the end, he **was dancing** with his sister.

Todd was at a wedding reception last night.  He **ate** cake and **drank** wine.  He **toasted** the bride and groom and in the end, he **danced** with his sister.

8. Bob was at a wild party last night.  He **was drinking** pints of beer and **doing** shots.  He **was wearing** a lamp shade on his head and **singing** crazy songs with friends.

Bob was at a wild party last night.  He **drank** pints of beer and **did** shots.  He **wore** a lamp shade on his head and **sang** crazy songs with friends.

9. Fred was in the attic yesterday afternoon.  He **was opening** up boxes.  He **was looking** at wedding photos and old mementos.  After that he **was reading** his sister's diary.

Fred was in the attic yesterday afternoon.  He **opened** up boxes.  He **looked** at wedding photos and old mementos.  After that he **read** his sister's diary.

10.

Eric stayed home from school yesterday.  He **was watching** TV and **playing** video games in the morning.  In the afternoon, he **was taking** a nap.  In the evening, he **was playing** pool.

Eric stayed home from school yesterday.  He **watched** TV and **played** video games in the morning.  In the afternoon, he **took** a nap.  In the evening, he **played** pool.

11.

Jeff was at his restaurant yesterday. At lunch he **was waiting** on tables and **helping** the chef.  He **was interviewing** new dishwashers and paying the bills.

Jeff was at his restaurant yesterday. At lunch he **waited** on tables and **helped** the chef.  He **interviewed** new dishwashers and paid the bills.

12.

Mark was at the park this morning.   He **was hanging** out at the basketball court and **passing** the ball around.  He **was shooting** baskets and he **was keeping** score.

Mark was at the park this morning.   He **hung** out at the basketball court and **passed** the ball around.  He **shot** baskets and he **kept** score.

13.

George was in his dorm room last night.  He **was listening** to the radio and **checking** his email.   Then he **was doing** some laundry.  He **was avoiding** his homework.

George was in his dorm room last night.  He **listened** to the radio and **checked** his email.   Then **he did** some laundry.  He **avoided** his homework.

14.

Bob was in his lab last night.  He **was analyzing** data and **setting** up a new experiment.  He **was listening** to music and **feeling** animated.

Bob was in his lab last night.  He **analyzed** data and **set** up a new experiment.  He **listened** to music and **felt** animated.

15.

Last night Dave was downtown.  He **was sitting** on a bench and **listening** to his I-pod.  He **was chilling** out and **avoiding** his homework.

Last night Dave was downtown.  He **sat** on a bench and **listened** to his I-pod.  He **chilled** out and **avoided** his homework

16.

Last night Michael was in his new car.  He was **speeding** down highway 99 and **listening** to his favorite CD.  He was **singing** along and **thinking** about his girlfriend.

Last night Michael was in his new car.  He **sped** down highway 99 and **listened** to his favorite CD.  He **sang** along and **thought** about his girlfriend.

17.

Last night Paul was in the kitchen.  He **was preparing** a meal for his family.  First he **was steaming** vegetables and **cooking** some rice.  Then he **was grilling** fish.

Last night Paul was in the kitchen.  He **prepared** a meal for his family.  First he **steamed** vegetables and **cooked** some rice.  Then he **grilled** fish.

18.

Yesterday Sam was in the library.   He **was studying** for his bio exam.  He **was reviewing** his notes and **reading** his textbook.  He **was memorizing** the key terms.

Yesterday Sam was in the library.   He **studied** for his bio exam.  He **reviewed** his notes and **read** his textbook.  He **memorized** the key terms.

19.

Yesterday Nathan was at a peace rally.  He **was marching** down the road and **talking** to other students.  He **was holding** up a sign.  Also, he **was passing** out pamphlets.

Yesterday Nathan was at a peace rally.  He **marched** down the road and **talked** to other students.  He **held** up a sign.  Also, he **passed** out pamphlets.

20.

Yesterday Edward was at Costco.  He **was checking** out the electronics section.  He **was sampling** food and **picking** up some school supplies.   Then he **was trying** on sunglasses.

Yesterday Edward was at Costco.  He **checked** out the electronics section.  He **sampled** food and **picked** up some school supplies.   Then he **tried** on sunglasses.

21.

Last night Bill was at a birthday party.  He **was drinking** some beer and **meeting** new friends.  After midnight, he **was guzzling** vodka and **popping** balloons.

Last night Bill was at a birthday party.  He **drank** some beer and **met** new friends.  After midnight, he **guzzled** vodka and **popped** balloons.

22.
Yesterday Carlo was at a BMW dealership.  He **was planning** to buy a used car, but he **was test-driving** some new model.  In the end, he **was signing** on the dotted line and **driving** a new car off the lot.

Yesterday Carlo was at a BMW dealership.  He planned to buy a used car, but he **test-drove** a new model.  In the end, he **signed** on the dotted line and **drove** a new car off the lot.
